# Diagnosis, treatment, and prognosis of stage IB non-small cell lung cancer with visceral pleural invasion

**DOI:** 10.3389/fonc.2023.1310471

**Published:** 2024-01-15

**Authors:** Zegang Ruan, Xin Zhuo, Chenyang Xu

**Affiliations:** Department of Thoracic Surgery, Ganzhou People’s Hospital, Jiangxi Medical College, Nanchang University, Ganzhou, Jiangxi, China

**Keywords:** non-small cell lung cancer, stage IB, visceral pleural invasion, adjuvant chemotherapy, treatment

## Abstract

With the increasing implementation of early lung cancer screening and the increasing emphasis on physical examinations, the early-stage lung cancer detection rate continues to rise. Visceral pleural invasion (VPI), which denotes the tumor’s breach of the elastic layer or reaching the surface of the visceral pleura, stands as a pivotal factor that impacts the prognosis of patients with non-small cell lung cancer (NSCLC) and directly influences the pathological staging of early-stage cases. According to the latest 9th edition of the TNM staging system for NSCLC, even when the tumor diameter is less than 3 cm, the final T stage remains T2a if VPI is present. There is considerable controversy within the guidelines regarding treatment options for stage IB NSCLC, especially among patients exhibiting VPI. Moreover, the precise determination of VPI is important in guiding treatment selection and prognostic evaluation in individuals with NSCLC. This article aims to provide a comprehensive review of the current status and advancements in studies pertaining to stage IB NSCLC accompanied by VPI.

## 1 Introduction

As per the latest Global Burden of Cancer report released by the International Agency for Research on Cancer in 2022, the estimated number of new malignant cases worldwide in 2020 was around 19 million, with approximately 10 million cancer-related deaths. Lung cancer stands as one of the primary contributors to cancer mortality, accounting for roughly 18% of all cancer-related deaths. It is projected that the global incidence of new cancer cases will reach 28.4 million by 2040 (1). With the increasing focus on early lung cancer screening and physical examinations, the detection rate of early-stage lung cancer has witnessed a continuous rise. Presently, lung cancer remains the most prevalent and lethal malignancy in the world (1). Therefore, early detection, diagnosis, and treatment play a vital role in patient prognosis. However, there is still ample room for further lung cancer prevention and treatment advancements.

The TNM staging system for non-small cell lung cancer (NSCLC) has undergone multiple revisions since its inception in the 1960s, with accurate staging playing a crucial role in prognostic assessment and treatment decision-making. Although there is room for further improvement in precise staging, it is widely recognized as a critical foundation for prognostic evaluation and treatment guidance. Rami-Porta et al. (2) proposed comprehensive revisions to T staging, elucidating the objectives of these revisions and highlighting the value and significance of T staging in guiding precise treatment for NSCLC. They emphasized the importance of distinguishing between visceral pleural invasion (VPI) and parietal pleural invasion. Numerous previous studies have demonstrated that VPI is associated with more aggressive disease behavior and poor prognostic factors (3–6).

According to the 8th edition of the TNM staging system for NSCLC by the Union for International Cancer Control (UICC) and the American Joint Committee on Cancer (AJCC), even if the maximum diameter of the primary tumor is ≤3 cm, the presence of VPI leads to an upstaging of T-stage to T2a, resulting in the transition from stage IA to stage IB in terms of pathological staging (7). Currently, there is a contentious debate regarding the optimal approach to surgical resection and postoperative adjuvant therapy for patients with stage IB NSCLC exhibiting VPI, with inconsistent guidelines on this matter. This article aims to comprehensively review the current status and progress of research about the diagnosis, treatment, and prognosis of stage IB NSCLC with VPI.

## 2 Visceral pleural invasion

### 2.1 Definition of visceral pleural invasion

The pleura is comprised of two layers: the visceral pleura, which envelops the surface of the lungs, and the parietal pleura, which adheres to the inner surface of the chest wall. These layers create a closed pleural cavity (8). This article focuses on the structure of the visceral pleura, specifically exploring its characteristics in diseases.

Histologically, the visceral pleura can be divided into five layers: the mesothelial layer, the submesothelial connective tissue layer, the outer elastic layer, the inner elastic layer, and the deep connective tissue layer, in sequential order from outermost to innermost (9). VPI is defined as the infiltration and penetration of tumor tissue through the elastic layer of the visceral pleura, which also encompasses invasion of the pleural surface (10–13). To avoid confusion with pathological tumor staging (pTNM), some researchers have proposed using the abbreviation “PL” instead of “P” to denote the pleura (13).

### 2.2 Diagnosis of visceral pleural invasion

The detection and assessment of VPI in NSCLC can be accomplished through various methods. Preoperative chest computed tomography (CT) images provide essential information for diagnosing VPI. Certain CT image features, such as pleural retraction and pleural indentation, enhance the accuracy of early detection of VPI in peripheral NSCLC (14, 15). Onoda et al. (16) conducted a study to evaluate the correlation between pleural markers on CT scans and VPI, involving 221 patients with peripheral NSCLC who underwent surgical resection. The labeling of pleural abnormalities on CT scans improved the predictive precision of VPI when the tumor was ≤3 cm in diameter and did not directly contact the pleural surface. Moreover, clinicopathological characteristics like age, histological type, and tumor differentiation level can serve as additional indicators for diagnosing VPI (17). A prediction model that combines preoperative CT findings with pathological features yields a more accurate prognosis of VPI (18).

**Figure 1:**
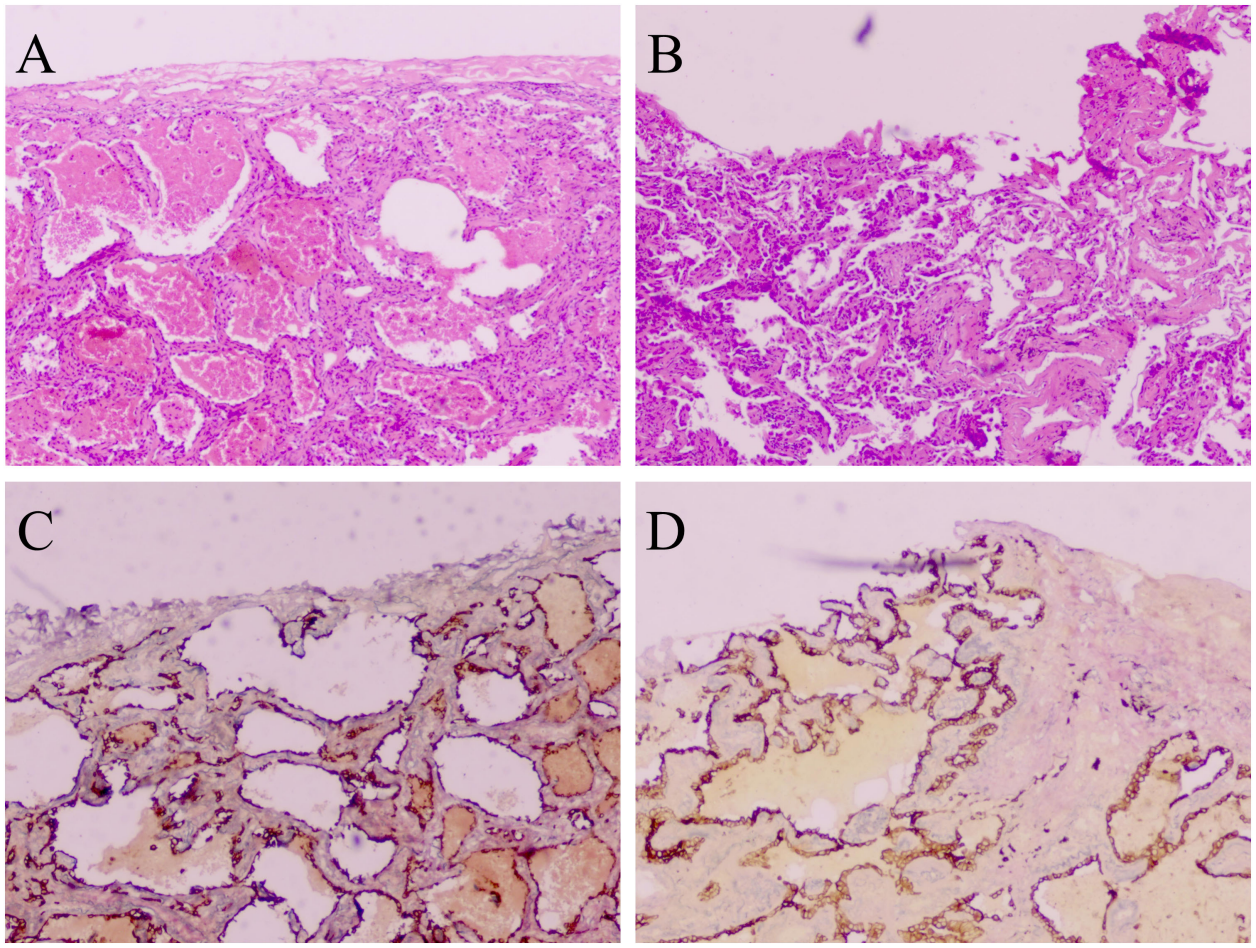
VPI is confined to the elastic layer without penetration of the pleural surface (PL1), stained with hematoxylin and eosin **(A)**. VPI involving penetration of the pleural surface (PL2), stained with hematoxylin and eosin **(B)**. VPI is confined to the elastic layer without penetration of the pleural surface (PL1), stained with elastic staining **(C)**. VPI involving penetration of the pleural surface (PL2), stained with elastic staining **(D)**. A-D: 40 × magnification.

Studies have demonstrated that the use of white light patterns in combination with autofluorescence can enhance the sensitivity and accuracy of intraoperative diagnosis of VPI during thoracoscopic surgery (19). However, relying solely on intraoperative visualization may lead to significant errors in determining VPI, necessitating postoperative histopathology for confirmation. Conventional hematoxylin and eosin staining may occasionally present challenges in clearly distinguishing between PL0 and PL1 stages, and it may even miss instances of tumor penetration through the elastic fiber layer. Elastic staining plays a crucial role in providing significant prognostic insights and facilitating accurate pathologic staging in cases where pleural invasion remains undetected by 10% hematoxylin and eosin staining (20). Common techniques for elastic staining include Victoria blue, Gomori’s aldehyde-fuchsin, or Weigert’s resorcin-fuchsin (21). some researchers advocate for the utilization of elastic staining assays to aid in identifying the extent of disruption in the elastic fiber layer (13, 22–24) (**Figures 1A-D**). Furthermore, recent findings have demonstrated that the utilization of dual-block elastin staining can effectively identify patients with more pronounced VPI (25).

### 2.3 Grading of visceral pleural invasion

In 1988, Hammar introduced a grading system for VPI in his book ‘Pulmonary Pathology’ (26). Subsequently, the Japanese Lung Cancer Society widely adopted Hammar’s grading scheme. However, when the sixth edition of the UICC/AJCC TNM staging system for NSCLC was released in 1997, although VPI was recognized as a crucial factor for improved staging, Hammar’s classification was not used to precisely define it (27).

As a result, the seventh edition of the TNM staging system for NSCLC in 2009 proposed the ‘Modified Hammar classification’. The modified Hammar classification categorizes the extent of VPI as follows: p0 indicates no breach of the elastic fiber layer by the tumor; p1 signifies tumor infiltration beyond the elastic lamina without exposure of the pleural surface; and p2 denotes tumor invasion of the pleural surface of the visceral layer, specifically the mesothelium (28). This grading criterion for VPI has been in use since then. However, in a study by Warth et al. (29) involving 173 NSCLC patients without lymph node metastasis, no significant differences were observed when analyzing survival rates based on the degree of VPI alone. This suggests that assessing the degree of VPI solely based on the elastic fiber layer may not be sufficient.

**Figure 2:**
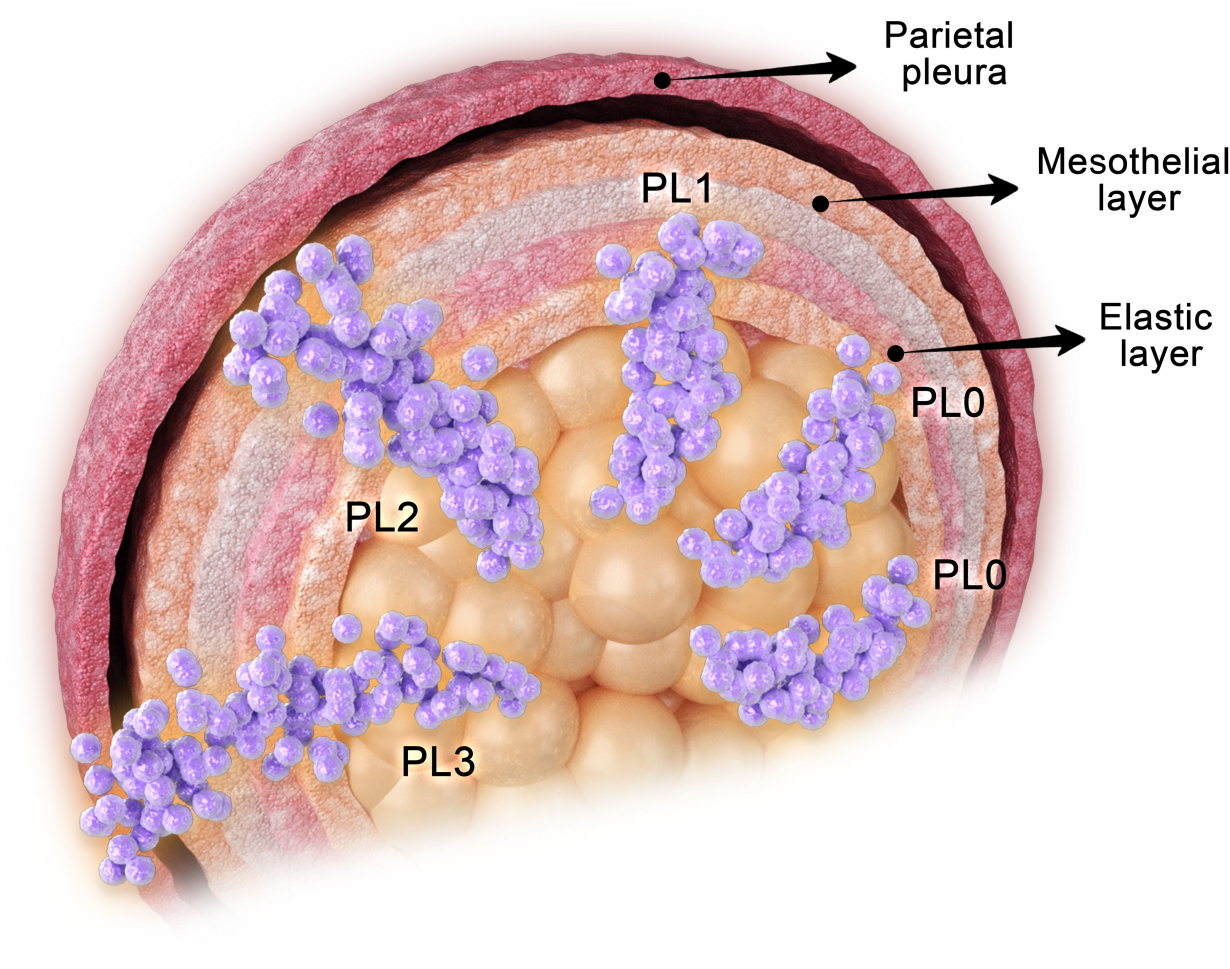
Grading of VPI in NSCLC.

Overall, the extent of VPI impacts the prognosis following resection of NSCLC, and in clinical practice, it is recommended to categorize VPI into three grades: PL0, PL1, and PL2 (30) (**Figure 2**).

## 3 Visceral pleural invasion and tumor staging

Accurate staging of NSCLC is crucial for the effective treatment and prognostic management of patients. VPI plays a significant role in the unfavorable prognosis of NSCLC and is a crucial component of the staging system (10, 22, 31, 32). Precise staging of early-stage NSCLC relies heavily on the accurate assessment of VPI (22). In the sixth edition of the staging system, any tumor that invaded the visceral pleura was classified as T2 (27). However, the seventh edition, proposed in 2009, focused more on detailed tumor size thresholds and did not adequately address the adverse effect of VPI (28). The 8th edition of the TNM staging system for NSCLC classified tumors as T2a when their diameter was less than 3 cm but accompanied by VPI (7).

It is important to note that researchers have gradually recognized the risk factor of VPI. Before the release of the seventh edition of the staging system, Shimizu et al. (11) concluded from a retrospective analysis of 1653 patients with T1, T2, and T3 NSCLCs that tumors with a diameter greater than 3 cm and VPI should be upgraded to T3 status. Some scholars have also proposed an improved classification for the pT2 category: T2a (tumor diameter 3-5 cm and P0-1) and T2b (tumor diameter >5 cm or P2) (33). However, these recommendations were not adopted in the seventh edition of the staging system. In 2007, Hung et al. (34) conducted a retrospective analysis of 445 resected patients with stage I NSCLC with a diameter less than 3 cm. The study concluded that VPI did not significantly affect the overall and disease-free survival (DFS)rates. Therefore, they suggested that NSCLC with VPI of less than 3 cm in diameter should be categorized as T1 rather than T2.

Before the release of the 8th edition of the staging system for NSCLC, Nitadori et al. (35) conducted a study that revealed no significant correlation between VPI and prognosis in patients with tumor diameters smaller than 2 cm. However, a significant correlation was observed in patients with tumor diameters ranging from 2 to 3 cm. Kudo et al. (36) recommended that the subsequent version of the TNM staging system for NSCLC classify tumors with diameters of 3.1-5 cm, accompanied by VPI, as T2b. Furthermore, some scholars have proposed that T1 tumors with VPI should be upgraded to T2a, T2a tumors with VPI should be classified as T2b, and T2b tumors with VPI should be categorized as T3 (37, 38). In 2012, Fibla et al. (39) conducted a survival analysis of 289 patients with stage IB (T2aN0M0) NSCLC according to the seventh edition of the AJCC classification. They divided the patients into three groups based on tumor size and the presence of VPI. The study revealed that patients with stage IB tumors measuring more than 3 cm but less than or equal to 5 cm, along with VPI, had a significantly worse prognosis compared to patients with “T2a” tumors solely based on VPI or tumor size. Therefore, it is recommended to upgrade these patients from the current IB stage to stage IIA. Shim et al. (12) suggested upgrading the T stage to T3 when the tumor diameter exceeds 5 cm but is less than 7 cm and accompanied by VPI, aligning with the eighth edition of the T staging system.

According to the eighth edition of the staging system for NSCLC, tumors with a diameter of 3 cm or less that invade the visceral pleura are classified as stage T2 (7). Yang et al. (6) conducted a study in 2017 that supported this staging system, suggesting that early-stage tumors with diameters less than 3 cm and VPI should be classified as a higher T stage. Furthermore, their findings indicated that tumors with diameters greater than 3 cm should be classified into higher T stages. However, some researchers and scholars have proposed that, in the absence of further large-scale multicenter studies on NSCLC, there is no need to upgrade patients with VPI and a maximum tumor diameter of 3 cm or less to the T2 stage (40).

In addition, Qi et al. (41) suggested considering escalation to the pT2b stage for early-stage NSCLCs with a diameter between 3.1 and 4.0 cm accompanied by VPI. Cai et al. (42) reached a comparable finding through an analysis of an extensive patient database comprising individuals with early-stage non-small cell lung cancer. Their conclusion suggests that for this specific population, characterized by a diameter of less than 3 cm, it should be classified as a T2a stage, exhibiting a more favorable prognosis. The relationship between the degree of VPI and tumor stage has been extensively explored in numerous relevant studies. A retrospective study revealed that in patients with NSCLC without lymph node metastasis and with a tumor diameter of 2 cm or less, tumors with PL1 features should be classified as stage T1, while tumors with PL2 features should be classified as stage T2 (43). T2a-PL2 tumors were categorized as stage T2b, indicating a poorer prognosis (44). Another study reached a similar conclusion that for tumors of 3 cm or less in NSCLC, tumors with PL1 features should still be classified as stage T1 rather than T2 (45). However, a different perspective exists for lymph node-negative NSCLC of 3 cm or less: there is a belief that patients with PL1 features should be classified as stage pT2a to improve staging accuracy (46).

To summarize, the staging of tumors in these patients should involve a comprehensive assessment of both tumor diameter and the extent of VPI, to develop an integrated staging approach. Particularly for patients with tumor sizes below 3 cm, along with concomitant VPI (PL1), it might be suitable to categorize them within a stage exhibiting a more favorable prognosis. We anticipate further studies to contribute additional evidence for the subsequent iteration of TNM staging for NSCLC.

## 4 Visceral pleural invasion and the nature of the nodule

Extensive research has demonstrated a substantial association between NSCLC with the presence of VPI and the characteristics of the nodules. In patients who underwent resection for stage I NSCLC, the ground glass component has been identified as a more reliable prognostic indicator (47). However, in cases of mixed ground-glass nodules, VPI did not serve as a prognostic predictor (48). Among patients with solid nodules, those without VPI exhibited a more favorable prognosis compared to individuals with pure glass nodules and mixed ground-glass nodules. Notably, the study did not further analyze the subgroups of PL1 and PL2 about VPI (47).

In a retrospective analysis of CT imaging involving 115 patients with pure ground-glass nodules, Zhao et al. (49) discovered that lung adenocarcinomas appearing as pure ground-glass nodules were typically not accompanied by VPI. Furthermore, multiple studies have revealed that VPI impacts the prognosis of patients with solid nodules, although its significance may vary among certain individuals with solid nodules (48, 50, 51). While VPI can be observed in both pure and mixed ground-glass nodules, it is more frequently observed in ground-glass nodules with a diameter exceeding 2 cm. Additionally, PL2 invasion is less common in patients with ground-glass nodules (52).

## 5 Visceral pleural invasion and lymph node involvement

In exploring the connection between VPI tumors and lymph node involvement, researchers have made notable contributions. Zhang et al. (53) discovered that among 740 peripheral upper lobe lung tumors, only 7 exhibited lower mediastinal lymph node metastasis, and all of these 7 tumors were characterized by VPI. VPI stands as a significant prognosticator for lymph node metastasis in clinical stage IA NSCLC (54). The optimal extent of lymph node clearance varies depending on the presence of VPI, with VPI(+) tumors necessitating a more extensive lymph node dissection compared to VPI(-) tumors of the same T1 size (55). However, it has also been demonstrated that the existence of VPI does not demonstrate a substantial correlation with lymph node involvement (56).

In summary, both VPI and lymph node involvement serve as indicators of unfavorable tumor prognosis. Although limited evidence exists regarding the relationship between the two, further studies are anticipated in the future.

## 6 Stage IB and surgical approach

Surgical resection remains the preferred treatment option for patients diagnosed with stage I and II NSCLC (57). Extensive research has been conducted on the various approaches to surgical resection in early-stage NSCLC cases involving VPI.

In 1995, Martini et al. (58) examined 598 patients with stage I NSCLC who underwent surgical resection and discovered that sublobar resection led to higher recurrence rates and lower survival rates, regardless of the histologic type. However, Koike et al. (59) conducted a retrospective analysis of 328 patients with clinical stage IA NSCLC who underwent wedge resection or segmental resection. They found that segmental resection could be the preferred sublobar resection approach for clinical stage IA NSCLC, specifically for patients with tumors lacking definite pleural invasion. In 2022, Song et al. (60) conducted a propensity-matched analysis and competing risk analysis of 2,717 cases of NSCLC surgical modalities. They identified lobectomy as the preferred surgical approach for NSCLC cases with VPI. Furthermore, due to the higher incidence of lymph node metastasis in tumors with VPI, more extensive lymph node dissection is required (36). Recent retrospective studies have reported that in early-stage NSCLC cases with tumors ≤3 cm in diameter and VPI, lobectomy outperforms sublobar resection in terms of survival prognosis (61, 62).

However, certain studies have indicated that for stage IB NSCLC, the prognosis of sublobar resection is comparable to that of lobectomy. In cases where VPI is discovered after segmental resection in patients with stage I NSCLC, continuing with lobectomy is unlikely to yield additional survival benefits (63, 64).

## 7 Stage IB and adjuvant chemotherapy

Adjuvant chemotherapy (ACT) treatment regimens incorporating platinum-based chemotherapeutic agents as the primary component have demonstrated enhanced prognostic outcomes for overall survival (OS) in patients with completely resected NSCLC. Furthermore, they have shown improved survival rates in individuals with early-stage NSCLC who exhibit high-risk factors for recurrence (65–69). In comparison to the seventh edition of the TNM staging system for NSCLC, the eighth edition of the staging system better identifies those patients with early-stage NSCLC who would benefit from platinum-based ACT, particularly those classified as stage II (70). However, the utilization of ACT in patients with stage IB NSCLC remains a topic of considerable controversy. In recent years, numerous clinical studies have investigated the potential benefits of ACT for patients with stage IB NSCLC (**Table 1**).

**Table 1:** Summary of studies on postoperative adjuvant chemotherapy in stage IB NSCLC.

Author	Year	Study design	Interventions	Outcomes
Park et al. (68)	2013	retrospective	adjuvant group (n = 60) vs. observation group (n = 59)	5-year OS: 88.2% vs. 64.7%, p = 0.01; 5-year DFS: 74.0% vs. 51.3%, p = 0.01
Tsutani et al. (69)	2022	retrospective	adjuvant group (n = 222) vs. observation group (n = 418)	5-year OS: 92.7% vs. 81.7%, p < 0.0001; 5-year RFS: 81.4% vs. 73.8%, p = 0.023
Wang et al. (70)	2019	retrospective	adjuvant group (n = 137) vs. observation group (n = 265)	5-year OS: 82.4% vs. 87.6%, p = 0.021
Park et al. (71)	2018	retrospective	adjuvant group (n = 27) vs. observation group (n = 62)	DFS: p = 0.662, OS: p = 0.866
Xu et al. (72)	2022	retrospective	adjuvant group (n = 55) vs. observation group (n = 55)	DFS: p = 0.13
Li et al. (73)	2019	retrospective	adjuvant group (n = 196) vs. observation group (n = 196)	OS,DFS: p > 0.05
Xie et al. (74)	2020	retrospective	adjuvant group with VPI (n = 145) vs. observation group with VPI (n = 951)	OS: p = 0.216
Lee et al. (74)	2023	retrospective	3 cm < tumor <=4 cm: adjuvant group (n = 573) vs. observation group (n = 262)	OS: p < 0.001, CSS: p = 0.001
Lee et al. (75)	2023	retrospective	3 cm < tumor <=4 cm: adjuvant group (n = 573) vs. observation group (n = 262)	OS: p < 0.001, CSS: p = 0.001

NSCLC, non-small cell lung cancer; VS, versus; OS, overall survival; DFS, disease-free survival; RFS, recurrence-free survival; VPI, visceral pleural invasion; CSS, cancer-specific survival.

Relevant research has indicated that VPI is a significant prognostic factor for stage IB NSCLC. However, even in high-risk patients, ACT has shown no impact on the prognosis of stage IB NSCLC (71). Furthermore, studies have demonstrated that ACT may not contribute to improved DFS in individuals with completely resected stage IB NSCLC (72, 73).

Incorporating a database of stage IB NSCLCs from 2010 to 2015, Xie et al. (74) conducted a multifactorial analysis using Cox proportional risk regression. Their findings revealed that even in patients with VPI, ACT did not confer benefits to those with stage IB NSCLC. Additionally, a study by Lee et al. (75) observed that ACT improved OS in patients with stage IB lung adenocarcinoma and tumor diameters larger than 3 cm but ≤4 cm. However, for patients with tumor diameters ≤3 cm and VPI, ACT did not contribute to a survival advantage. It has been reported that excessive use of ACT can be avoided in patients with lymph node-negative NSCLC and tumor diameters of ≤3 cm (45). In conclusion, ACT did not significantly enhance long-term survival in patients with stage IB NSCLC accompanied by VPI.

Significantly, emerging evidence suggests that the presence of VPI is an independent prognostic factor associated with unfavorable outcomes in patients diagnosed with stage I NSCLC. Consequently, a more aggressive therapeutic approach, such as ACT, may be warranted for individuals presenting with VPI (76–79). Specifically, ACT could be a viable treatment option for patients with stage IB NSCLC, particularly those with high-risk factors including VPI, vascular invasion, advanced age, and poorly differentiated tumors (80, 81). Moreover, ACT has demonstrated the potential to enhance long-term prognosis in patients with stage IB NSCLC who have a history of previous malignancies (82).

In the case of lymph node-negative NSCLC, the combined influence of VPI and tumor size exhibits a synergistic effect on survival. A study conducted by Zhang et al. (83) found no significant association between ACT and improved 5-year survival in patients diagnosed with stage IB-IIA NSCLC. However, for patients with tumor diameters ranging from 3 to 4 cm and concurrent VPI, ACT may confer potential benefits. A similar conclusion was reached by Wightman et al. (84), suggesting that ACT should be particularly considered for patients with tumor diameters of 3-4 cm and the presence of VPI. Additionally, ACT has been reported to enhance survival in a substantial cohort of T2N0M0 patients who underwent complete resection, with significant outcomes observed in patients with tumors measuring less than 4 cm in diameter (85). It is worth noting that ACT is recommended for patients with stage IB disease with VPI, particularly when the tumor diameter is ≥4 cm (86, 87).

Building upon these studies, Hou et al. (88) conducted a comprehensive investigation in 2022 involving 1050 patients with NSCLC characterized by pathologic T2N0M0 staging. The patients were divided into two groups: those who received ACT and those who did not. The results indicated that ACT only improved OS in patients with tumor diameters exceeding 4 cm (OS: P = 0.003; DFS: P = 0.013). Furthermore, ACT exhibited a significant 5-year survival benefit in patients with wild-type epithelial growth factor receptor (EGFR) (P = 0.022). Therefore, in patients with tumor diameters greater than 4 cm and the presence of wild-type EGFR, ACT may offer a survival advantage. Additionally, in patients diagnosed with N0 NSCLC, PL2 emerges as a crucial prognostic factor closely associated with recurrence and poorer overall survival, thereby justifying the consideration of ACT (89).

Nevertheless, further investigation is required to fully comprehend the significance of ACT in patients presenting with VPI. Consequently, there is an imperative to develop relevant prognostic markers and models to identify patients who may not necessitate adjuvant therapy and to identify those who could benefit from such treatment. Numerous factors have been assessed, including VPI, which may influence the selection of ACT. However, the majority of currently available data are retrospective, limiting the applicability of these markers in present clinical practice (90).

Some scholars have identified risk factors influencing postoperative recurrence and have constructed predictive models for recurrence, aiming to inform the selection of ACT for stage IB NSCLC patients (91–96). However, relatively limited research has been conducted on predictive models for postoperative recurrence in patients with stage IB NSCLC featuring VPI. Ren et al. (97) developed a scale that provides personalized predictions of recurrence-free survival following resection in patients with stage I lung adenocarcinoma. High-risk patients with a score ≥245 may derive benefits from postoperative ACT. Similarly, the identification of >4 circulating tumor cells defines a high-risk subgroup, offering a novel strategy for optimal clinical decision-making in stage IB lung adenocarcinoma (98). Furthermore, nomograms offer more accurate prognostic predictions for patients with resected stage IB NSCLC (81). ACT has demonstrated superior OS compared to non-ACT in patients with stage IB NSCLC. Nomogram modeling provides individualized predictions of OS in patients following surgical resection. Patients with tumor sizes ranging from 2 cm to 4 cm may represent potential candidates for adjuvant chemotherapy (99).

## 8 Stage IB and targeted therapy

In recent years, extensive research has been conducted on adjuvant targeted therapy following surgery for early to mid-stage NSCLC. Targeted therapy, overall, offers significant advantages over chemotherapy and is better tolerated by patients. A global multicenter phase III double-blind study known as ADAURA (100) has confirmed the considerable extension of DFS in patients with EGFR mutation-positive NSCLC (stages IB to IIIA) through the use of osimertinib. Notably, stage IB patients treated with osimertinib experienced a remarkable 60% reduction in the risk of disease progression or death. Another phase II clinical study (GASTO1003, CORIN), led by Wang et al. (101), involved 128 surgically resected stage IB patients (based on the 7th edition of TNM staging). The patients were randomly assigned to either the icotinib group (63 patients) or the observation group (65 patients). The icotinib group received one year of targeted therapy with icotinib, commencing six weeks after the surgery, while the observation group underwent observation until disease progression or intolerable adverse effects. The study concluded that postoperative adjuvant icotinib therapy significantly prolonged DFS by three years in patients with completely resected stage IB NSCLC harboring EGFR mutations. These findings were consistent when applying the 8th edition of the TNM staging system.

In summary, the CORIN study aligns with the results of the ADAURA trial, providing further evidence of the efficacy and tolerability of adjuvant EGFR-TKI as a postoperative treatment for patients with stage IB NSCLC with EGFR mutations.

## 9 Stage IB and immunotherapy

In the past, some studies related to immunotherapy for early-stage NSCLC have been conducted, but there are almost no studies on immunotherapy for patients with stage IB with VPI. In terms of neoadjuvant immunotherapy, the Checkmate 816 (102) study revolutionized the field of neoadjuvant immunotherapy by demonstrating that combining three cycles of preoperative nivolumab with chemotherapy significantly increased the rate of pathological complete remission compared to chemotherapy alone. Similarly, the keynote671 (103) study revealed that neoadjuvant pembrolizumab, combined with chemotherapy before resection, followed by adjuvant pembrolizumab, led to significant improvements in event-free survival, major pathologic response, and pathological complete remission when compared to neoadjuvant chemotherapy alone followed by surgery. Turning to adjuvant immunotherapy, Felip et al. (104) conducted the IMpower010 study, a randomized phase 3 trial, which demonstrated that adjuvant immunotherapy with atezolizumab, following adjuvant chemotherapy, significantly enhanced DFS in patients with completely resected stage II-IIIA NSCLC. This study offered a promising therapeutic option for surgically resected early-stage NSCLC patients. However, it did not provide detailed survival data specifically for stage IB. Subsequent findings from the randomized phase III clinical trial PEARLS/KEYNOTE-091 indicated that pembrolizumab substantially improved DFS compared to placebo, potentially serving as a novel therapeutic approach for stage IB-IIIA NSCLC after complete resection (105).

Overall, numerous studies have demonstrated the advantages of immunotherapy; nevertheless, the current research on immunotherapy for stage IB patients remains limited. Furthermore, although previous studies addressed this stage, their classification was based on the 7th edition of the TNM staging system. According to the 8th edition, the majority of stage IB patients should be reclassified as stage IIA.

## 10 Visceral pleural invasion and prognosis and survival

Extensive research has been conducted to investigate the relationship between VPI and prognosis. One study indicated that VPI does not significantly impact OS in patients with early-stage NSCLC (106). Building upon this finding, Seok et al. (107) conducted a retrospective study in 2017, examining the survival of 90 patients with N0 NSCLC and concurrent VPI. Ultimately, they concluded that the extent of VPI may not affect the prognosis of N0 NSCLC patients who undergo surgical resection with VPI. However, this study has certain limitations, including a small sample size and a short postoperative follow-up period, which may introduce errors in the results.

On the contrary, Oyama et al. (108) held a different perspective. They included 1,488 patients with NSCLC who underwent surgical resection and examined the extent and location of VPI. By comparing various clinicopathological factors, they found a significant difference in the prognosis of patients with PL0 and PL1-3 tumors. Additionally, they observed no difference in prognosis among the PL1, PL2, and PL3 groups. However, it was also demonstrated that patients with PL0 and PL1 degrees of VPI exhibited similar survival rates, suggesting that these two groups could be considered negative for VPI, while the PL2 group had an impact on patient survival (56).

In 2015, Adachi et al. (109) further identified, through a retrospective study, that VPI influenced postoperative survival in patients with NSCLC with N0 or N1 metastases, rather than the extent of invasion. Building upon previous studies, David et al. (110) analyzed 1166 patients with pN0M0 NSCLC who underwent lobectomy, including 214 patients with VPI and 952 patients without VPI. The results revealed that the impact of VPI on survival varied based on tumor size, with weak correlations between VPI and OS or DFS in tumors smaller than 5 cm.

Regarding stage IB NSCLC patients with concurrent VPI, differences in tumor location did not affect patient prognosis (111). Additionally, total pleural adhesions were not found to be a risk factor for recurrence after lobectomy for stage I NSCLC with VPI (112). A study by Ahn et al. (113) revealed that percutaneous lung puncture biopsy did not significantly increase the risk of pleural recurrence in stage I NSCLC; however, VPI was identified as the cause of pleural recurrence.

## 11 Limitations and prospects

VPI is a significant negative prognostic factor in NSCLC. Despite numerous studies conducted on this topic, there are still limitations and opportunities for improvement. For tumors with a diameter of less than 3 cm, the presence of VPI leads to a final pathological stage of IB. Although studies have examined the extent of VPI, the impact of different degrees of invasion on patient prognosis and tumor staging for small tumors remains unclear. Moreover, there is a lack of research on tumor penetration of the inner and outer elastic layers of the visceral pleural, which hampers our understanding of the relationship between these two levels of invasion and prognosis. Therefore, further data collection is necessary.

The existing studies on this subject have yielded varied results, emphasizing the need for comprehensive investigations in the future. Such studies should consider multiple factors, including VPI, histological type of NSCLC, tumor differentiation, nodule characteristics, lymph node involvement, inflammatory markers, tumor gene mutations (such as EGFR gene mutations), and the degree of VPI. By collectively exploring the impact of these factors on the survival prognosis of this patient population, we can gain a more comprehensive understanding.

Furthermore, accurate determination of VPI is crucial. While elastic staining is widely acknowledged as a diagnostic method for VPI, its utilization in actual pathology remains relatively uncommon, and there is no consensus on the choice of staining method. Considering the importance of VPI in guiding clinical staging and postoperative adjuvant therapy, a combination of elastic staining and immunohistochemistry can be considered for diagnosing VPI.

## 12 Summary

In conclusion, VPI plays a pivotal role in the unfavorable prognosis of individuals diagnosed with NSCLC. The accurate identification of VPI holds the utmost importance in determining prognostic outcomes and guiding therapeutic interventions for this condition. To precisely evaluate the influence of VPI on the survival and prognosis of patients with stage IB NSCLC, a comprehensive examination of various factors, including tumor characteristics, surgical approaches, and the nature of the nodule, is warranted. However, previous studies have predominantly focused on the prognostic impact of VPI alone, neglecting the exploration of comprehensive factors encompassing tumor-related aspects, surgical techniques, and the degree of VPI. We strongly believe that future large-scale multicenter prospective studies will enable a more accurate prediction of prognosis in patients with stage IB NSCLC featuring VPI.

## Author contributions

ZR: Writing – original draft. XZ: Writing – original draft. CX: Supervision, Writing – review & editing.
